# Investigating the experiences of New Zealand MRI technologists: Exploring intra-orbital metallic foreign body safety practices

**DOI:** 10.1002/jmrs.26

**Published:** 2013-11-19

**Authors:** Philippa K Jacobs, Suzanne Henwood

**Affiliations:** 1 River Radiology, Victoria Clinic750 Victoria Street, Hamilton, Waikato, New Zealand; 2 Unitec – Medical ImagingUnitec Ratanui Street Henderson, Auckland, New Zealand

**Keywords:** Intra-orbital metallic foreign body, lived experiences, MRI technologist, safety practices

## Abstract

**Introduction:**

Qualitative research is lacking regarding the experiences of magnetic resonance imaging (MRI) technologists and their involvement in workplace safety practices. This article provides a gateway to explore, describe and document experiences of MRI technologists in New Zealand (NZ) pertaining to intra-orbital metallic foreign body (IMFB) safety practices.

**Methods:**

This phenomenological study describes the experiences of seven MRI technologists all with a minimum of 5 years' NZ work experience in MRI. The MRI technologists were interviewed face-to-face regarding their professional IMFB workplace experiences in order to explore historical, current and potential issues.

**Results:**

Findings demonstrated that aspects of organization and administration are fundamentally important to MRI technologists. Varying levels of education and knowledge, as well as experience and skills gained, have significantly impacted on MRI technologists’ level of confidence and control in IMFB practices. Participants’ descriptions of their experiences in practice regarding decision-making capabilities further highlight the complexity of these themes. A model was developed to demonstrate the interrelated nature of the themes and the complexity of the situation in totality.

**Conclusions:**

Findings of this study have provided insight into the experiences of MRI technologists pertaining to IMFB safety practices and highlighted inconsistencies. It is hoped that these findings will contribute to and improve the level of understanding of MRI technologists and the practices and protocols involved in IMFB safety screening. The scarcity of available literature regarding IMFB safety practices highlights that more research is required to investigate additional aspects that could improve MRI technologists’ experiences.

## Introduction

Although magnetic resonance imaging (MRI) has been utilized in the clinical setting for over 25 years, there has been limited research of MRI technologists' experiences pertaining to intra-orbital metallic foreign body (IMFB) safety practices. MRI is an important clinical tool but can be harmful to the patient if an IMFB is present. Although the incidence is not known, IMFBs are of particular concern because any slight movement can result in blindness. Safety guidelines have been widely published; however, there is a lack of knowledge about how well such guidelines are implemented in practice and how useful they are. The MRI technologist is usually the gatekeeper for MRI safety; therefore, their experiences pertaining to IMFB safety practices could help assess the efficacy of current IMFB safety procedures and/or provide recommendations for improvements. Available resources regarding various lived experiences of radiographers appear to be limited to areas of advanced practice, radiographic reporting and other specific roles in radiography.

Previous studies conducted mainly in the United States of America (USA) have compared patient safety screening methods used in practice^1^ in order to create safety screening questionnaire prototypes that incorporate the espoused ‘best features’ from all models available (p. 198).^2^ As a result, MRI technologists' lived experiences have been overlooked regarding IMFB safety practices, and although the prevalence of IMFB injury is unknown, this study effectively addresses IMFB safety practices, from the MRI technologist's viewpoint.

A phenomenological study was conducted to provide greater insight and understanding of the lived experiences of seven MRI technologists actively involved in IMFB safety screening practices in New Zealand (NZ). The study provided a rich description of their experiences, roles and processes relating to their IMFB screening patients and captured the essence of the phenomena from their perspective.

## Methods

Prior to the study's commencement, research approval was obtained from the Unitec Research Ethics Committee (UREC). The study was performed in accordance with UREC's ethical standards, and the duration of participant recruitment and data collection occurred between March and April 2012. Although the research was carried out by a master's student as part of thesis research, the entire process was closely supervised, including review of transcriptions and findings throughout.

Experts in phenomenological studies suggest the inclusion of no more than 10 participants due to large volumes of data generated in that style of research.^3^ Therefore, seven actively employed and practicing MRI technologists within MRI institutions were selected: two male and five female participants (to reflect the current gender mix in the profession). Participants were required to have a minimum of 5 years' full-time equivalent NZ MRI work experience. All participants provided informed consent prior to involvement.

Hycner (1999, cited in Groenewald)^4^ states that ‘the phenomenon dictates the method (not vice-versa) including even the type of participants' (p. 156). Therefore, purposive sampling was utilized for selecting participants for this study which Welman and Kruger^5^ consider to be the most effective method of non-probability sampling. It allowed the researcher to intentionally select individuals who were able to reflect on and be willing to share experiences regarding the phenomenon that were in concordance with the objectives of the study.^6^ Participant selection focused on seven individuals who could ‘tell their story’ coherently. The researcher determined this through the participants' forthright, honest and spontaneous responses to the topic at hand. However, had a chosen participant not been forthcoming or provided limited insight, inclusion of another participant would have been considered. This did not transpire.

A pilot study carried out on 10 individuals to develop the final data collection tools identified that geographical location within NZ was of no apparent significance regarding IMFB safety screening experiences, thus opening up the option for sampling across the whole country. However, due to time restrictions, location of the researcher and provision for face-to-face interviews, seven participants were selected from a spread of MRI technologists within the North Island of NZ only. The small sample of participants focused on gaining valid, rich descriptions of their experiences, rather than generalizable results for the profession.

Formal, intensive, semi-structured, face-to-face interviews using clean language^7^ were carried out by the researcher to facilitate the collection of deep descriptive data. Dictaphones were used throughout all interviews to record participants' conversations. The interview guide, developed through the pilot study, included a combination of open-ended and closed questions, with the majority being open-ended to encourage depth of data from the participants' personal experiences. Closed questions were used to elicit factual and demographic data and included (but were not restricted to) questions regarding their MRI experience and previous MRI workplace locations. Open-ended questions explored the depth and understanding of participants' lived experiences regarding IMFB safety screening practices. Sentence starters for additional probing were also developed as part of the interview process to enable the researcher to maintain the focus of conversations and seek elaboration of explanations from participants when required. As a result, no repeat interviews were required. Participants were provided with a copy of their transcript for revision and no corrections were requested.

The process of analysis chosen for the phenomenological framework utilized a combination of Colaizzi's and Fade's methods, which employ components of both descriptive and interpretive methods.^3^,^8^ These methods were most compatible with this particular research and effective with small sample groups of up to 10 respondents as it enables the researcher to write up single cases while exploring themes shared between cases.^9^

Abstraction and designating themes is a challenging process that requires substantial interaction with the transcripts.^3^ Fade states that it is important that the names given to the events, objects, actions and interactions in the data reflect the true context of the participants' words.^3^ This process enabled a better understanding of the participants' recollections of experiences.

Descriptions of the participants' experiences were then grouped according to the identified themes. An overall description and understanding of IMFB safety practice experiences was constructed through typifications demonstrating collective themes of experiences that occurred across the group of participants as well as unique themes experienced by one or the minority of participants.

### Participant demographics

The participants' work experience ranged from 5 to 15 years, with an average of 8.6 years' experience in MRI. All had completed postgraduate diplomas in health science (PGDipHSc) specializing in MRI, which is the minimum eligibility requirement for registration in NZ and a prerequisite to gaining a full scope of practice in MRI. Three of the participants had undertaken further postgraduate study and gained master's degrees.

The participants' MRI work experience identified that four had undertaken all their clinical experience in NZ and three had gained additional MRI experience overseas, one having worked in South Africa and two in the United Kingdom. The total number of years of workplace experience outside of NZ totalled 9.5 years among the three with overseas experience, with an average of 3.2 years.

In addition, all participants had experience working in more than one MRI department, with four being the maximum number of departments and 2.4 the average.

## Results

**Figure 1:**
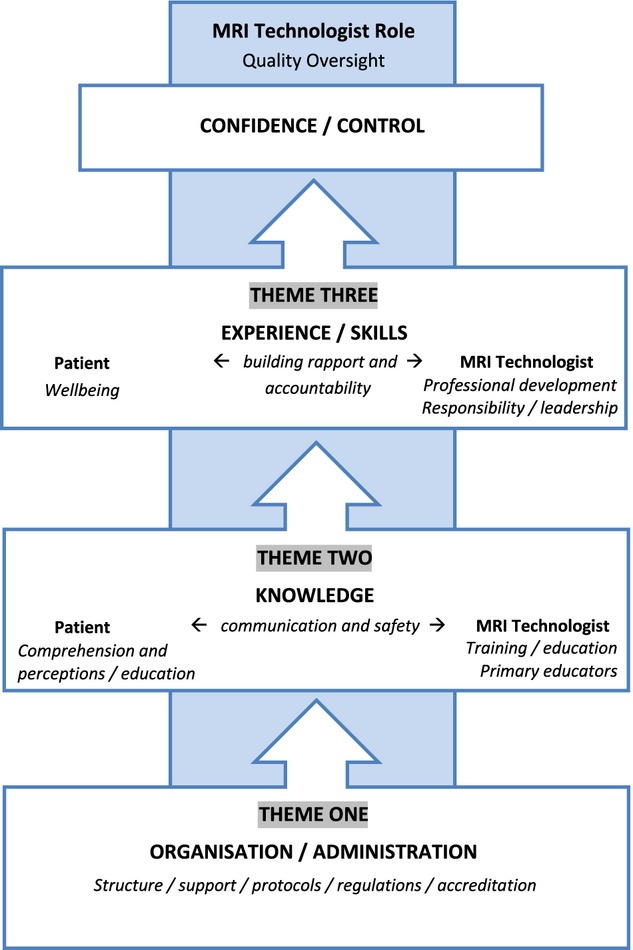
Interactions pertaining to MRI technologists' lived experiences in relation to intra-orbital metallic foreign body safety practices.

Figure 1 provides a basic overview of the data findings and identifies four key factors pertaining to the participants' experiences in relation to IMFB safety screening. The diagram also indicates that organization/administration fundamentally underpins the progression of interaction between these factors, impacting on knowledge and experience/skills which ultimately affects levels of confidence and control. Furthermore, there is a strong central MRI technologist role (shaded background) which influences and provides fundamental support for effective IMFB safety practices; it is central and pivotal to each theme. Discussion of the study's findings demonstrates the implications on IMFB safety practices when this role is not effectively supported and equipped. Table 1 provides participant quotes to justify the associated themes identified in Figure 1.

**Table 1:** Themes and associated quotes from study participants.

**Theme one – organization/administration**	
Structure and support	‘We try and pick them up early enough through receptionist questioning. If they're picked up, they come in and have it done before they come in [for the booked MRI scan]’. – Participant 1 ‘Where you learn MR affects your standard of practice, because if you started somewhere that wasn't as good at screening, you may be more lax about it and I'm sure there are people that way inclined’. – Participant 2
Protocols	‘That's why we have the three screening sessions … sometimes you don't remember things the first time around’. – Participant 3 ‘… some [of the IMFB safety screening protocols being used] are not evidenced based practices … I think that's why different places have different policies’. – Participant 4 ‘I think it's been 5 years since they were written … I guess, “Because it's always been done this way”, is not good enough in a court of law’. – Participant 3
Regulations and accreditation	‘It is very interesting because no one has said who actually is responsible for this [MRI safety]. I would have thought that as a governing body they [IANZ] should be’. – Participant 1
**Theme two – knowledge**	
*MRI technologist's* training and education	‘I must admit that working here [New Zealand], and doing the postgraduate study has been great for me because all it was, was being shown how it works [at my previous workplace overseas]. There was no background … nobody taught you anything.’ – Participant 5
*MRI technologist's* primary educators	‘… I don't think they [patients] have a clue really’. – Participant 1 ‘It's an everyday issue, an every-patient issue …’. – Participant 6
*Patient* comprehension and perceptions/education	‘A lot of people seem to think that, “Oh it happened years ago, it's not relevant”. I often get that …’. – Participant 6 ‘They're [patients] very anxious about what's going on. It's the anxiety, the unknown …’. – Participant 2 ‘I think the education by their doctors is hopeless, absolutely hopeless’. – Participant 4
**Theme three – experience and skills**	
*MRI technologist's* professional development	‘I think it's a learned [experience] thing, to read people in this way’. – Participant 4 ‘To pick up on what's important and what isn't, it helps having a bit of experience’. – Participant 6
*MRI technologist's* primary educators	‘… If you keep asking, ask again, and then you eventually get the correct answer. You just have to talk to them …’. – Participant 5
*Patient* well-being	‘The big thing about your screening time with your patient is that it's your time to relax your patient … build a rapport and make the whole examination much easier for them’. – Participant 2 ‘We do our best to set up a rapport with patients’. – Participant 7
*MRI technologist's* confidence/control	‘I think the longer you do it, the more confident you get in making the right decision to x-ray or not … because you've heard so many variations [of patient stories]’. – Participant 6 ‘My first thing was developing the way I've now become [with screening patients] and that's what I do and I'm still doing it’. – Participant 7

Participants demonstrated that IMFB safety practices follow a layered, interactive process. The diagrammatic representation of the findings identifies three key themes regarding the role of MRI technologists relating to IMFB safety practices (Fig. 1). Participants perceived most IMFB safety screening processes to be effective in eliminating safety incidences; however, areas lacking understanding and communication between patients, administrative staff and MRI technologists appeared to impact on the effectiveness of the overall process.

This study demonstrates that at an organizational level, well-controlled IMFB safety practices in the workplace are an important element of MRI technologist workflow and patient safety. All participants indicated that three-way screening processes were helpful and a necessary part of IMFB safety practices. Only one participant identified negative aspects of administrative booking procedures due to an employer lacking knowledge. However, some participants acknowledged that further education of administrators is required, given that they are the first point of contact for patients in the screening process. The educational role of some referring clinicians was also found to be wanting.

Formal education regarding IMFBs was not offered to any participants, either by their employer or by the tertiary provider. Participants identified that their knowledge regarding aspects of IMFB safety practices, mainly the removal of IMFBs, was in most instances gained during patient interviews. Of concern is that as new MRI practitioners, the participants identified feeling uncertain and ill prepared regarding IMFB patient safety screening. This also highlighted implications for participants regarding the potential effects of negative socialization of safety practices and the impact on patient safety due to varied practices. Only one participant was offered a formal, 6-week introductory course in MRI covering safety aspects while working in the United Kingdom.

Participants identified frequently encountering patients who, in their view, lack comprehension regarding the examination being undertaken, impacting on their understanding of MRI safety as well as their cooperation with IMFB safety screening during MRI technologist interviewing. One participant indicated that this is largely due to patients not receiving sufficient information either from their referrer or the practice, and competitiveness between practices to service patients quickly. Furthermore, two participants indicated that some referrers are not sufficiently informed about MRI safety issues to adequately enlighten their patients.

Participants identified themselves as primary educators regarding MRI safety which can impact on their workload, yet they felt it was essential to ensure comprehensive, high-quality service, incorporating safety practices. This is indicative of MRI technologists’ positive and pivotal role across all themes in terms of IMFB safety practices. The challenges MRI technologists experience have demonstrated that patients often consider an IMFB injury to be inconsequential in MRI safety screening if the injury occurred many years ago. The participants believe referrers are partly to blame for not discussing IMFB injuries with patients and indicated the workload for radiographic screening could be reduced if it was done properly by referring clinicians, and quality of practice improved, if this was addressed. Furthermore, patients’ previous imaging histories, experiences and distorted media portrayal of MRI scanners were also considered to be responsible for compromising and, in some instances, falsely representing MRI. This has caused varying degrees of anxiety and, at times, uncooperative attitudes in some patients during IMFB screening.

Participants’ feedback demonstrated the importance of their role in terms of quality oversight of safety practices and maintaining high standards of patient care. While all participants involved in the study regarded patient care as a priority, there were instances where the MRI technologist's role of quality oversight had potentially been compromised due to ill-defined roles and responsibilities. Certain safety issues were also occasionally overlooked. The researcher believes that raising MRI technologists’ awareness of their significant role and diverse responsibilities could lead to a more autonomous profession, as previously mooted by NZ MRI technologists.^10^

Three varied radiographic protocols for assessing IMFBs with radiographic imaging were identified as follows:

1. Any prior injury requires an orbital radiograph.
2. More than two IMFBs removed require an orbital radiograph.
3. Patient deemed to be a poor historian requires an orbital radiograph.

Only one participant noted an increased clinical screening focus in recently published literature. The second protocol listed above was identified by two participants as being too interpretive. Participants’ experiences of protocol revision drew mixed reviews with five participants maintaining the need to be individually driven to assess new literature and update protocols. Regardless of this, the participants were generally happy with their current workplace radiographic protocols. However, it is noted that the second protocol referred to above has meant that two participants go outside their workplace radiographic protocol in order to feel confident in their personal screening practices and maintain patient safety – highlighting how some practitioners are already demonstrating a level of autonomous practice.

MRI technologists in this study indicated that confidence with IMFB safety practices increased with experience. Only after gaining experience in the field did the participants believe they developed techniques to build rapport and improve cooperation with patients. It was noted that the MRI technologists intuitively interpreted patient responses through listening and questioning techniques that develop over time. In addition, participants who received peer support and witnessed role modelling of good practice in the early stages of their training identified that such support was important.

As MRI technologists gain experience, the study's findings suggest that they need to be mindful of becoming overconfident or developing a blasé attitude regarding IMFB safety screening practices as this may impact on patient safety and colleagues’ workplace experiences. This concurs with views of Plous^11^ who claimed that ‘no problem in judgment and decision making is more prevalent and more potentially catastrophic than overconfidence’ (p. 217). Some of the participants acknowledged discomfort regarding this issue, but only one participant indicated it had been problematic in the workplace. The participants were not forthright in discussing the legal ramifications of accountability in such situations.

Despite MRI technologists expressing satisfaction with their IMFB safety practices, there were indications from some participants that some form of regulation may be beneficial. Participants referred to negative experiences regarding radiology accreditation in NZ and felt that assessment of safety should be considered and incorporated, potentially through a combination of existing regulatory bodies (International Accreditation NZ, National Radiation Laboratory, and/or New Zealand Institute of Medical Radiation Technology (NZIMRT), as indirectly indicated by some participants).

## Discussion

While the study findings did not identify any significant threats to patient safety based on current policies, in at least some workplaces, education regarding IMFBs was shown to be lacking in certain aspects. There is potential for more to be done to increase knowledge and overall competence in decision-making relating to MRI technologists, administrative staff, patients and referrers.

### MRI technologist and administrative staff education

Issues specific to lack of knowledge and the way in which this impacts on MRI technologists' decision-making implies that more education regarding IMFB injuries and their removal prior to clinical work is warranted. It is imperative that prospective MRI technologists enter into IMFB patient safety screening fully informed.

This study suggests that enhancing MRI technologists’ knowledge regarding IMFBs would be beneficial in order to minimize confusion and further support them in the clinical environment. These measures would ensure that IMFB safety practices are better aligned nationally and guarantee that MRI technologists have accurate knowledge in order to make informed screening choices.

Furthermore, this study highlighted that there may be merit in MRI technologists and receptionists carrying out safety training together, as this could further streamline cooperation and communication, and enhance understanding of each other's contribution/role. In addition, streamlined training may also ensure that the same messages are being conveyed.

This study recognized that the education and support provided through a peer learning/mentoring scheme at qualification level would be helpful in encouraging and assisting training MRI technologists and aiding those who are still developing screening accuracy. It is difficult to ascertain how and when this knowledge is generated if not through mentoring and effective role modelling. This study has identified that training MRI technologists require appropriate mentoring in order to develop basic competencies regarding IMFB safety practices.

### Patient education

Patient education was the area of most concern to participants. While they identified themselves as the primary patient educators, it is recommended that clinicians could provide their patients with more information prior to referring them for MRI examinations. At present, this appears to be lacking.

One participant suggested emailing or faxing information to patients prior to their arrival for a scan as a method of information distribution could be an effective alternative to posting it. Popularity of email technology has grown rapidly alongside the rise of the Internet.^12^ Sachoff^13^ maintains that attitudes have changed because of technology and as a result ‘organizations need to understand these trends if they want to reach their customers exactly where and how they would like to be reached’ and that ‘businesses must stay ahead of the trends’ (para. 4).

The MRI technologists’ role in IMFB safety practices was shown to be pivotal to the safe use of MRI technology. However, there were instances in which participants had not always ‘led the way’ and where IMFB screening processes had been undermined or failed. This was demonstrated when issues were identified and MRI technologists did not address or rectify situations at hand. It is recommended that MRI technologists undergo leadership-style training as part of their postgraduate education to enable them to step up and be proactive in their role of quality control and lead more effectively. This can also apply to the preparation and education of patients prior to a procedure.

### Study limitations

It is recognized that as a result of the study's inclusion criteria, MRI technologists with considerable overseas experience may have inadvertently been excluded from the study. There was no way of knowing this as only NZ experience is indicated on the register of practitioners and it is an accepted possible limitation of this study, potentially limiting data to NZ experience. It is acknowledged, however, that some of the MRI technologists involved in the study had gained prior MRI experience working overseas and used this to supplement their NZ workplace experience, therefore offering additional insight and depth to the study and thus reducing the impact of any selection criteria limitations. The researcher also acknowledges that the study's results are not generalizable to all NZ MRI technologists due to the small sample size.

## Recommendations and Implications for Best Practice

Potential issues relating to MRI technologists working outside their scope of practice have been identified in this study. Further investigation is suggested regarding the support provided by NZ regulatory bodies (NZIMRT and NZ Medical Radiation Technologists Board) and what can potentially be done to reinforce the regulation of MR safety practices and raise MRI technologists’ awareness of their responsibilities when performing IMFB safety screening.

### Educational framework initiative

Implementation of a national, educational initiative would ensure foundational knowledge is gained and its effectiveness on MRI technologists’ experiences could be tested. This could impact on the confidence of those new to the profession and even have the potential to improve workplace mobility for MRI technologists, if standards existed. Furthermore, it is expected that this would have a positive impact on patient care.

The NZIMRT, recognized both nationally and internationally as representing the profession in NZ, could potentially accept more responsibility for updating and ensuring current MR safety compliance.^14^ ‘The institute encourages the exchange of information between individuals to help keep them at the leading edge of medical imaging’ (para. 2).^14^ The findings pertaining to MRI technologists, based on international knowledge and best practice, could then be communicated to them individually, prior to the NZIMRT's annual conference with an invitation to participate in a separate modality group discussion session as part of the conference. The majority of NZIMRT work is carried out by volunteers and this would require MRI technologists taking responsibility for instigating and leading the work. Charge radiographers would then be responsible for ensuring that the relevant information is also circulated and read by all the staff in their departments. However, this study has provided evidence that there are some MRI technologists who are not sufficiently motivated to instigate change.

A separate study investigating and evaluating levels of training provided to those performing the task of screening may be useful, especially for those ‘softer’ skills like intuition, and for trainees determining that they have all the information required to make a decision regarding a patient's safety.

### Improved patient information distribution methods

Potential improvements in patient education may be achieved by referring clinicians adopting a more proactive approach that is encouraging patients who express uncertainty to contact the prescribed radiology practice to discuss concerns regarding safety contradictions. This may require MRI technologists to allocate more time to educating patients prior to their appointments in order to improve patient knowledge and understanding, streamline workflow and reduce potential IMFB risks. In addition, the core role of MRI technologists has the potential to instil mutual confidence and control, and endorse the three key themes that the researcher identified (refer to Fig. 1) and is therefore highly recommended.

The introduction of an additional step after the referral may also be useful for enhancing patient knowledge that is the option to view videos (potentially through *YouTube* or cell phone applications) so that they can receive visual information prior to making the appointment or, having made the appointment, prior to arriving. It is recommended that embracing various forms of current technology to communicate with patients could more effectively relay information to patients. Furthermore, prompts could also be included in the information provided to patients, to contact the radiology department if ever having experienced an IMFB injury. Early contact with patients could potentially reduce patient ignorance.

## Conclusion

Despite the small sample of participants involved in this study, aspects of MRI technologists' experiences pertaining to IMFB safety practices have highlighted and demonstrated the aspects regarding successful patient screening by providing insight into the following:

- the complexity and pivotal role of MRI technologists,
- aspects of participants' experiences and attitudes pertaining to and impacting on safety practices,
- improving MRI technologist, administrative staff and patient education,
- streamlining the clinical screening process,
- challenges pertaining to decision-making regarding IMFB safety practices.
